# Sodium-Calcium Exchange in Intracellular Calcium Handling of Human Airway Smooth Muscle

**DOI:** 10.1371/journal.pone.0023662

**Published:** 2011-08-15

**Authors:** Venkatachalem Sathish, Philippe F. Delmotte, Michael A. Thompson, Christina M. Pabelick, Gary C. Sieck, Y. S. Prakash

**Affiliations:** 1 Department of Physiology and Biomedical Engineering, Mayo Clinic, Rochester, Minnesota, United States of America; 2 Department of Anesthesiology, Mayo Clinic, Rochester, Minnesota, United States of America; Johns Hopkins School of Medicine, United States of America

## Abstract

Enhanced airway contractility following inflammation by cytokines such as tumor necrosis factor alpha (TNFα) or interleukin-13 (IL-13) involves increased intracellular Ca^2+^ ([Ca^2+^]_i_) levels in airway smooth muscle (ASM). In ASM, plasma membrane Ca^2+^ fluxes form a key component of [Ca^2+^]_i_ regulation. There is now growing evidence that the bidirectional plasma membrane Na^+^/Ca^2+^ exchanger (NCX) contributes to ASM [Ca^2+^]_i_ regulation. In the present study, we examined NCX expression and function in human ASM cells under normal conditions, and following exposure to TNFα or IL-13. Western blot analysis showed significant expression of the NCX1 isoform, with increased NCX1 levels by both cytokines, effects blunted by inhibitors of nuclear factor NF-κB or mitogen-activated protein kinase. Cytokine-mediated increase in NCX1 involved enhanced transcription followed by protein synthesis. NCX2 and NCX3 remained undetectable even in cytokine-stimulated ASM. In fura-2 loaded human ASM cells, NCX-mediated inward Ca^2+^ exchange as well as outward exchange (measured as rates of change in [Ca^2+^]_i_) was elicited by altering extracellular Na^+^ and Ca^2+^ levels. Contribution of NCX was verified by measuring [Na^+^]_i_ using the fluorescent Na^+^ indicator SBFI. NCX-mediated inward exchange was verified by demonstrating prevention of rising [Ca^2+^]_i_ or falling [Na^+^]_i_ in the presence of the NCX inhibitor KBR7943. Inward exchange-mode NCX was increased by both TNFα and IL-13 to a greater extent than outward exchange. NCX siRNA transfection substantially blunted outward exchange and inward exchange modes. Finally, inhibition of NCX expression or function blunted peak [Ca^2+^]_i_ and rate of fall of [Ca^2+^]_i_ following histamine stimulation. These data suggest that NCX-mediated Ca^2+^ fluxes normally exist in human ASM (potentially contributing to rapid Ca^2+^ fluxes), and contribute to enhanced [Ca^2+^]_i_ regulation in airway inflammation.

## Introduction

In airway smooth muscle (ASM), regulation of intracellular Ca^2+^ ([Ca^2+^]_i_) involves Ca^2+^ release from and reuptake into the sarcoplasmic reticulum (SR), as well as plasma membrane Ca^2+^ influx and efflux [1], [2], [3], [4], [5], [6], [7], [8], [9]. In ASM, following stimulation with agonists, Ca^2+^ influx is known to occur through both voltage-gated [10] and receptor-gated [11] channels. Furthermore, controlled Ca^2+^ influx in response to agonist-induced SR Ca^2+^ depletion occurs [5], [6], [12], [13], [14], which helps replenish intracellular Ca^2+^ stores (store-operated Ca^2+^ entry, SOCE). Following [Ca^2+^]_i_ elevation, mechanisms to reduce Ca^2+^ levels are activated. In this regard, SR Ca^2+^ reuptake and plasma membrane Ca^2+^ ATPase are major mechanisms. However, an additional mechanism that received relatively little attention is the bidirectional Na^+^/Ca^2+^ exchanger (NCX).

A role for NCX in [Ca^2+^]_i_ regulation of cardiac muscle is well-established [15], [16], [17]. In the forward, efflux or outward exchange mode, NCX uses the energy within the trans-membrane Na^+^ gradient to exchange 1 Ca^2+^ for 3 Na^+^ (electrogenic). Efflux mode NCX in cardiac muscle is widely accepted [16], [18], [19]. Reverse, or influx mode NCX also occurs under certain conditions in cardiac muscle, as tested using inhibitors such as KBR7943 [16], [18], [19]. In smooth muscle, early studies provided evidence for NCX-mediated tone development in aortic smooth muscle, followed by numerous reports suggesting that NCX could contribute to Ca^2+^ influx and contraction in vascular smooth muscle [20], [21], [22]. The physical proximity of NCX to perimembranous SR [23], [24], and a relationship between NCX and TRPC proteins [22], [25], [26] indicate a role for NCX in [Ca^2+^]_i_ homeostasis.

In ASM, NCX has been reported to participate in [Ca^2+^]_i_ regulation in cow [27], [28], pig [29], and guinea pig [30], but apparently not at all in dog [31], [32]. Whether NCX participates in [Ca^2+^]_i_ regulation of human ASM has been barely examined. Recently, NCX-mediated Ca^2+^ influx was reported in human ASM, apparently linked to SR Ca^2+^ store depletion via the regulatory protein STIM1 [33]. Assuming that Ca^2+^ fluxes via NCX are present in human ASM, this would represent a potentially rapid mechanism for regulating SR Ca^2+^ content in opposing ways: 1) providing Ca^2+^ via influx mode, increasing [Ca^2+^]_i_ and facilitating SR refilling; or 2) removing Ca^2+^ via efflux mode, decreasing [Ca^2+^]_i_, and SR refilling. Accordingly, a major goal of the present study was to establish the importance of influx vs. efflux modes of NCX in human ASM.

It is well-recognized that altered [Ca^2+^]_i_ regulation is a key component of the pathophysiology of airway diseases such as asthma [34] and chronic obstructive pulmonary disease [35] where increased expression of inflammatory cytokines such as tumor necrosis factor-alpha (TNFα) and the interleukins (IL) IL-1β and IL-13 enhance ASM contractility [36]. While the list of cytokines potentially involved in asthma is long, both TNFα and IL-13 have been the focus of considerable investigation. TNFα increases agonist-induced [Ca^2+^]_i_ and contractility of ASM including in humans [6], [36], [37], [38]. In a previous study [6], we demonstrated that TNFα increases SOCE in human ASM cells [6]. Similarly, in mouse and rabbit trachea, IL-13 enhances agonist-induced airway contractility [39]. Based on these previous studies, we selected TNFα and IL-13 to examine the effect of inflammation on NCX in human ASM. Given the apparent relationship between NCX and SOCE [33], we hypothesized that TNFα and IL-13 increase the influx mode of NCX in human ASM, thereby enhancing [Ca^2+^]_i_ levels as well as enhancing Ca^2+^ availability to refill the SR (and thus greater Ca^2+^ for agonist responses). In the present study, we used real-time imaging of [Ca^2+^]_i_ and intracellular Na^+^ ([Na^+^]_i_) in human ASM cells to determine NCX fluxes under normal conditions and with exposure to TNFα and IL-13. Pharmacological inhibitors and siRNA were used to inhibit NCX expression or activity to determine the importance of this mechanism in [Ca^2+^]_i_ regulation.

## Materials and Methods

### Isolation of Human ASM Cells

The techniques for isolation of human bronchial smooth muscle cells have been previously published [4]. Briefly, bronchi were obtained from surgical lung tissue of patients undergoing thoracic surgery at Mayo Clinic Rochester. Lung resection samples were incidental to patient surgery (typically lobectomies and pneumenectomies) and were always those samples that are discarded by the pathologist following diagnosis. The Institutional Review Board (IRB)-approved protocols allowed for initially review of patient histories, followed by complete de-identification of samples for storage and subsequent usage. In this study, we used airways from both males (4) and females (2). However, for the purposes of this study, no tissues were from smokers, asthmatics or patients with COPD, since we did not want to confound the effect of variable chronic inflammation and/or smoke exposure on what we considered “controls” and “cytokine exposed” samples. Instead, with confirmation by the surgical pathologist, we identified and used the normal areas of airways from patients with focal disease that underwent lung surgery (e.g. squamous cell carcinoma, granuloma etc., but not bronchoalveolar carcinoma or small cell carcinoma which tend to be widespread).

The process to obtain these de-identified samples, as well as all related studies using such samples, were reviewed, approved and stated as being not Human Subjects Research by the IRB of the Mayo Clinic, Rochester, MN. Accordingly, patient consent was waived.

Bronchioles were initially placed in Hanks' balanced salt solution (HBSS; Invitrogen) with 2.5 mM extracellular Ca^2+^, freed of cartilage, epithelium and surrounding tissues, and ASM cells isolated using collagenase and papain. Cells were plated in sterile culture flasks and grown in a 95% air/5% CO_2_ humidified incubator using DMEM F/12 supplemented with 10% FBS. All experiments were performed in cells prior to the 3^rd^ passage of subculture. In subsets of samples, ASM phenotype was verified by Western blots for smooth muscle actin and myosin, and agonist receptors, as well as lack of fibroblast markers. Cell viability was tested by exclusion of Trypan blue.

### Western Blot Analysis

Proteins were separated by SDS-PAGE (Criterion Gel System; Bio-Rad, Hercules, CA; either 10% or 4-15% gradient gels) and transferred to polyvinylidene fluoride (PVDF) membranes (Bio-Rad) for 60 min. Membranes were blocked for 1 h with 5% milk in TBS containing 0.1% Tween (TBST) and then incubated overnight at 4°C with anti-NCX antibodies (Santa Cruz). Following three washes with TBST, primary antibody was detected using horseradish peroxidase-conjugated secondary antibody and signals developed by Supersignal West dura Chemiluminescent Substrate (Pierce Chemical Co., Rockford, IL). The membranes were probed with monoclonal α-smooth muscle actin (Sigma) as loading control.

### Protein knockdown by siRNA

ASM at 60% confluence were transfected using 50 nM NCX1 siRNA (human SLC8A1; Ambion, 5′-CUA UCA UAG CUG AUC GGU Utt-3′) or negative control siRNA, Ambion, 5′-GCG CGC UUU GUA GGA UUC G-dTdT-3′ or TRPC3 siRNA (human TRPC3; Ambion, 5′-GGA CUC UAA AGG ACA UAU Utt-3′) with Lipofectamine 2000 (Invitrogen) as transfection agent in DMEM F/12 lacking FBS. Fresh growth medium was added 6 h after transfection and the cells analyzed after 48 h. The efficacy of siRNA knockdown was verified by Western analysis of decreased NCX1 protein expression. Both Lipofectamine (vehicle) and negative siRNAs were used as controls.

### Cytokines and drugs exposure

As previously described [8], [40], ASM cells were exposed for 24 h to either medium alone (control), 20 ng/ml recombinant human TNFα (Calbiochem), or 50 ng/ml recombinant human IL-13 (Calbiochem). ASM cells were exposed to SN50 (NF-κB inhibitor; 20 µM) or PD98059 (MEK1/2/ERK1/2 mitogen-activated protein (MAP) kinases inhibitor; 5 µM: Calbiochem) for 1 h prior to TNFα or IL-13 exposure (in the continued presence of inhibitor). In separate set of studies, cells were exposed to actinomycin D (transcription inhibitor; 1 µg/ml: Sigma) or cycloheximide (protein synthesis inhibitor; Sigma) in the presence of cytokines,

### [Ca^2+^]_i_ imaging

The techniques for [Ca^2+^]_i_ imaging of human ASM cells using fura-2 have been previously described [4]. Briefly, ASM cells plated on 8-well Labteks were incubated in 5 µM fura-2 AM (Invitrogen) for 60 min at room temperature and visualized with a fluorescence imaging system (MetaFluor; Universal Imaging, Downingtown, PA) on a Nikon Diaphot inverted microscope. In previous studies, we have used HBSS for examining [Ca^2+^]_i_. However, due to the need for additionally altering the Na^+^ concentrations for examining NCX, the current study was conducted using Tyrodes' solution [17]. Cells were initially perfused with 2 mM Ca^2+^ Tyrodes and baseline fluorescence established. A custom-built fluid level controller allowed cell perfusion with rapid exchange of perfusate (<300 ms). [Ca^2+^]_i_ responses of at least 10 cells per chamber were obtained. Fura-2-loaded cells were alternately excited at 340 and 380 nm and emissions at 510 nm collected separately at 1 Hz (Cascade 1 K 12-bit camera, Roper Scientific, Tucson, AZ). Results were expressed using the ratio of the 340 nm/380 nm wavelengths. Quantification of [Ca^2+^]_i_ levels was performed from fura-2 levels using previously described calibration procedures [4], [41].

### [Na^+^]_i_ imaging

Other investigators have previously reported using the ratiometric cell-permeant dye SBFI/AM (Invitrogen) for real-time measurement of [Na^+^]_i_ in cardiac muscle [42] and ureteral smooth muscle [43], [44]. The excitation/emission filters for fura-2 were also used for SBFI. A similar technique was used for ASM cells in this study. ASM cells were loaded for 60 min with 5 µM SBFI, and then washed with normal Tyrodes' solution. SBFI was detected using the fura2 filter sets as for [Ca^2+^]_i_ imaging. In a subset of preparations, calibration of [Na^+^]_i_ was performed *in vitro* by exposing SBFI-loaded ASM cells to different extracellular Na^+^ (in the additional presence of 10 µM gramicidin D and 100 µM strophanthidin or ouabain; data not shown). A mixture of two solutions with equal ionic strength in different proportions was used to vary extracellular Na^+^: one containing 145 mM Na^+^ (30 mM NaCl, 115 mM sodium gluconate) and zero K^+^, and the other 145 mM K^+^ (30 mM KCl, 115 mM potassium gluconate) but zero Na^+^. Solutions were buffered with 10 mM HEPES and additionally contained glucose and EGTA. Ca^2+^ and Mg^2+^ levels were maintained as in normal Tyrodes' solution [17].

### Determination of inward exchange (Influx) mode NCX

Control, TNFα-exposed and IL-13-exposed ASM cells were loaded with fura-2 (as above). Cells were perfused with normal Tyrodes (2 mM Ca^2+^/140 mM Na^+^) and baseline [Ca^2+^]_i_ measured [17]. Extracellular Ca^2+^ ([Ca^2+^]_o_) was then removed (0 Ca^2+^/140 mM Na^+^; zero-Ca Tyrodes) to “Na^+^-load” cells via activation of outward Ca^2+^ exchange mode NCX (reflected by decreased [Ca^2+^]_i_). Inward Ca^2+^ influx via L-type channels was inhibited using 1 µM nifedipine. Reduced [Ca^2+^]_i_ can result in Ca^2+^ release from the SR. Accordingly, SR Ca^2+^ release was simultaneously inhibited by exposing cells to 10 µM ryanodine (inhibiting Ca^2+^ release via ryanodine receptors) and 10 µM Xestospongin C (Xest-C; inhibiting IP_3_-mediated Ca^2+^ release). Thus, Na^+^ loading was achieved while preventing the activation of influx-mode NCX and SR Ca^2+^ dynamics. After 10 min, the perfusate was rapidly changed (<300 ms for complete replacement of bath solution) to 2 mM Ca^2+^/4 mM Na^+^ Tyrodes (zero-Na Tyrodes), activating inward exchange mode NCX. Nifedipine, ryanodine and Xest-C were maintained. To determine the mechanism underlying [Ca^2+^]_i_ changes, cells were pre-exposed to the NCX inhibitor KBR7943 (10 µM) in zero-Ca Tyrodes for 5 min prior to activation of inward exchange mode NCX by zero-Na Tyrodes in the continued presence of KBR7943.

In a second set of cells loaded with SBFI, [Na^+^]_i_ was directly measured. Cells underwent Na^+^-loading as above, followed by activation of inward exchange mode NCX by zero-Na Tyrodes. Given exchange of Na^+^ for Ca^2+^, [Na^+^]_i_ was expected to rise in zero-Ca Tyrodes, and rapidly fall with zero-Na Tyrodes. We used organic cation N-methyl-D-glucamine for Na^+^ replacement experiments. Specificity of NCX was verified by absence of falling [Na^+^]_i_ in the presence of KBR7943.

In third set of experiments, ASM cells transfected with NCX1 siRNA were loaded with fura2 or SBFI and [Ca^2+^]_i_ and [Na^+^]_i_, respectively, were measured using the protocols above.

To verify that the contribution of NCX-mediated Ca^2+^ inward exchange *per se* to the observed elevation in [Ca^2+^]_i_, two sets of additional studies were performed. In the first set, in ASM cells transfected with NCX1 siRNA, the extent of SOCE was evaluated using previously published protocols [5], [12]. In a second set of experiments, cells transfected with TRPC3 siRNA (we have previously shown that TRPC3 is a major mechanism mediating SOCE in human ASM [37]) were subjected to the protocol for evaluating inward exchange mode NCX.

### Determination of Outward exchange (Efflux) mode NCX

Following determination of baseline [Ca^2+^]_i_ levels in control, TNFα-exposed or IL-13-exposed ASM cells loaded with fura-2 in normal Tyrodes solution, the perfusate was changed to 0Ca^2+^/4 mM Na^+^ Tyrodes (zero-Na, zero-Ca Tyrodes) along with 10 µM cyclopiazonic acid (CPA; inhibitor of SR Ca^2+^ reuptake), which results in increased [Ca^2+^]_i_ without activation of either NCX mode (due to continued SR Ca^2+^ leak in the absence of reuptake). Some decrease in the plateau of the [Ca^2+^]_i_ response was expected due to eventual activation of the plasma membrane Ca^2+^ ATPase. When [Ca^2+^]_i_ has more or less reached a plateau, the perfusate was rapidly changed (<300 ms for complete replacement of bath solution) to zero-Ca Tyrodes, activating outward exchange mode NCX.

In a second set of experiments using SBFI-loaded cells, [Na^+^]_i_ was directly measured during activation of outward exchange mode NCX. Given the exchange of Na^+^ for Ca^2+^, [Na^+^]_i_ was expected to fall when exposed to zero-Na, zero-Ca Tyrodes, but rapidly increase when exposed to zero-Ca Tyrodes. Specificity of NCX was verified in NCX1 siRNA transfected cells.

### NCX and [Ca^2+^]_i_ Responses to Agonist

[Ca^2+^]_i_ responses to 10 µM histamine were evaluated in fura-2 loaded ASM cells (exposed to vehicle only, TNFα or IL-13) perfused with normal Tyrodes' solution. Peak [Ca^2+^]_i_ and the rate of fall of the response were measured. Following a thorough washout with Tyrodes' solution, cells were exposed to KBR7943, and [Ca^2+^]_i_ responses to histamine reevaluated in the presence of KBR7943. In a second set of experiments, [Ca^2+^]_i_ responses to histamine were compared in cells transfected with Lipofectamine alone, NCX1 siRNA or negative control siRNA.

### Statistical analysis

Six bronchial samples were used to obtain ASM cells. All biochemical and molecular biology experiments (e.g. western analysis, siRNA) were repeated at least 3 times, and *n* denotes the number of cells that were analyzed, although not all protocols were performed in each sample obtained. Drug effects on [Ca^2+^]_i_/Na^+^ responses were analyzed using ANOVA with a Dunnett's post hoc test.

## Results

### NCX Expression in ASM

**Figure 1:**
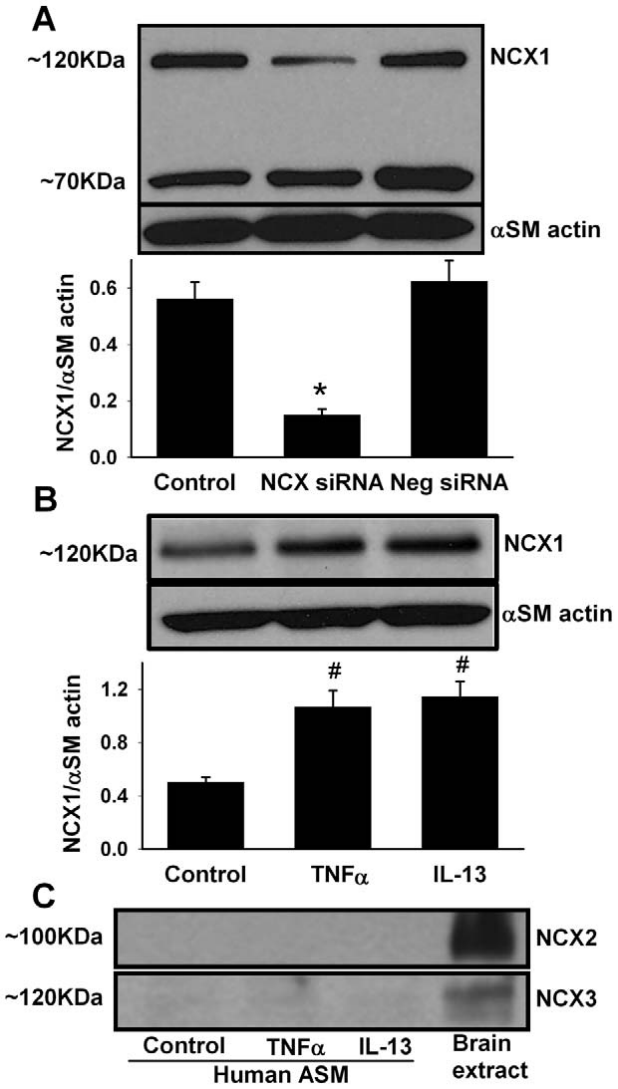
Effect of cytokines on expression of Na^+^/Ca^2+^ exchanger (NCX) protein isoform NCX1 in human airway smooth muscle (ASM). Under normal conditions, human ASM cells expressed only the NCX1 isoform, but not NCX2 or NCX3 (which were detected in positive controls, however). Transfection with small interference RNA (siRNA) targeting NCX1 resulted in significant reduction in protein expression (120KDa), while the vehicle (Lipofectamine) or scrambled siRNA (neg siRNA) had no significant effect. Overnight expression of ASM cells to the pro-inflammatory cytokines tumor necrosis factor alpha (TNFα) and interleukin-13 (IL-13) for 24 h substantially enhanced NCX1 (120KDa) expression compared to controls not exposed to cytokines. The other NCX isoforms remained undetectable. α-Smooth muscle (αSM) actin was used as loading control. Values are means ± SE (n = 5). * indicates significant NCX1 siRNA effect, # significant cytokine effect (p<0.05).

Western blot studies of human ASM cells showed significant expression of NCX1 protein (Figure 1A, 1B), with no expression of NCX2 or NCX3 (these isoforms were detectable in rat brain extract (positive control), verifying their absence specifically in ASM; Figure 1C). Protein bands for NCX1 were observed at 70 and 120 KDa (Figure 1A), but not at 160 KDa. Transfection of ASM cells with NCX siRNA substantially blunted NCX1 siRNA compared to Lipofectamine controls or scrambled siRNA controls which did not significantly influence NCX1 expression (Figure 1A; p<0.05 for specific siRNA effect).

Exposure to 20 ng/ml TNFα or 50 ng/ml IL-13 for 24 h significantly increased NCX1 protein expression in ASM (Figure 1B; p<0.05 on densitometric analysis for both cytokines). Only the 120 KDa band showed increased expression. Even with cytokine exposure, NCX2 and NCX3 remained undetectable in human ASM cells (Figure 1C).

**Figure 2:**
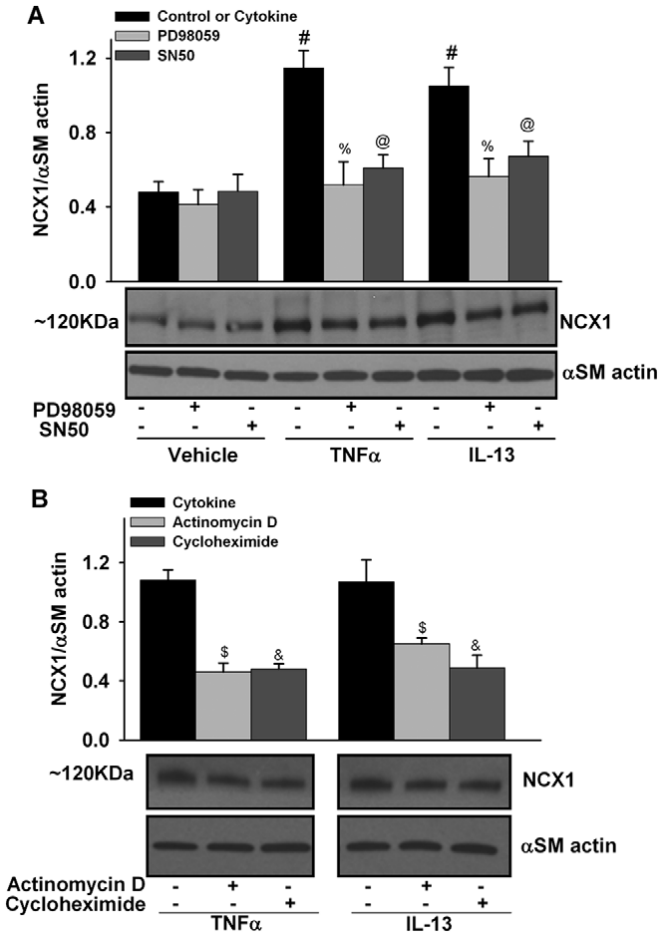
Mechanisms of cytokine-induced increase in NCX1 expression. A. Pre-exposure of ASM cells to 20 µM SN50 (NF-κB inhibitor) or 5 µM PD98059 (MEK1/2/ERK1/2 MAP kinase inhibitor) 1 h prior to TNFα or IL-13 exposure (24 h, in continued presence of inhibitor) blunted cytokine-induced increase in NCX1 expression (120KDa) compared to controls not exposed to cytokines. B. In separate sets of cells, pre-exposure of ASM cells to the protein synthesis inhibitor cycloheximide (1 µg/ml) or the transcription inhibitor actinomycin D (1 µg/ml) 1 hr prior to TNFα or IL-13 exposure (24 h, in continued presence of inhibitor) blocked NCX upregulation. αSM actin was used as loading control. Vehicle is culture media. Values are means ± SE (n = 4). # significant cytokine effect, % significant PD98059 effect, @ significant SN50 effect, $ significant actinomycin D effect, & significant cycloheximide effect (p<0.05).

To determine potential mechanisms by which NCX1 protein expression is enhanced by cytokines, ASM cells were pre-exposed to SN50 (NF-κB inhibitor; 20 µM: Sigma) or PD98059 (MEK1/2/ERK1/2 MAP kinase inhibitor; 5 µM: Calbiochem) for 1 h prior to TNFα or IL-13 exposure (in the continued presence of inhibitor). Subsequent Western analysis found that inhibition of either NF-κB or MEK1/2/ERK1/2 MAP kinases significantly blunted cytokine-induced increase in NCX1 (Figure 2A; p<0.05).

To investigate whether cytokine-induced increase in NCX1 was due to newly synthesized protein, the effects of the protein synthesis inhibitor cycloheximide and the transcription inhibitor actinomycin D were assessed (Figure 2B). Human ASM cells treated with cytokines in the presence of actinomycin D showed significantly reduced NCX expression, compared to cytokine exposure alone (Figure 2B; p<0.05). Inclusion of cycloheximide during cytokine treatment also significantly blocked NCX upregulation (Figure 2B; p<0.05). Together, these results suggest that increased NCX expression by cytokine exposure results from transcriptional activation and synthesis of additional NCX protein.

### Inward Exchange Mode NCX

**Figure 3:**
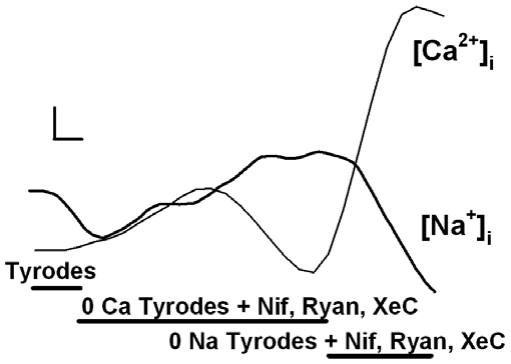
Evaluation of inward Ca^2+^ exchange mode NCX in human ASM cells using measurement of [Ca^2+^]_i_ (fura-2) vs. [Na^+^]_i_ (SBFI). Removal of extracellular Ca^2+^ in the presence of ryanodine (to inhibit SR Ca^2+^ release via ryanodine receptor channels), Xestospongin C (to inhibit release via IP_3_ receptor channels) and nifedipine (Nif; to inhibit L-type Ca^2+^ channels) resulted in Na-loading of ASM cells (illustrated by increased [Na^+^]_i_ levels in SBFI loaded cells). Following Na-loading, rapid re-introduction of extracellular Ca^2+^ and simultaneous removal of extracellular Na^+^ resulted in activation of inward Ca^2+^ exchange mode NCX, thus increasing [Ca^2+^]_i_ while decreasing [Na^+^]_i_. Horizontal scale bar is 10 s. Vertical scale bar is 50 nM for Ca^2+^ and 2 mM for Na^+^.

In fura-2 loaded control ASM cells perfused with normal Tyrodes solution (2 mM Ca^2+^/140 mM Na^+^), baseline [Ca^2+^]_i_ levels ranged between 80–140 nM (110 ± 10 nM). Following a slow increase in [Ca^2+^]_i_ levels was observed with removal of Ca^2+^ by perfusion with zero-Ca Tyrodes (with simultaneous SR inhibition; Figure 3). This was likely due to continued SR Ca^2+^ leak through channels not locked in the open state by ryanodine. Under these conditions, ASM cells were Na^+^-loaded for 10 min. Subsequently, rapid perfusion with zero-Na Tyrodes resulted in rapid increase in [Ca^2+^]_i_ levels (Figure 3). The rate of rise of [Ca^2+^]_i_ (a straight line through the steepest part of the curve within the first 5 s) ranged from 8.3 to 49.3 nM s^−1^ in control cells. Repetition of the inward exchange protocol in control experiments resulted in a<10% decrease in inward exchange rate. Accordingly, time-related bias in the inward exchange protocol was ignored.

**Figure 4:**
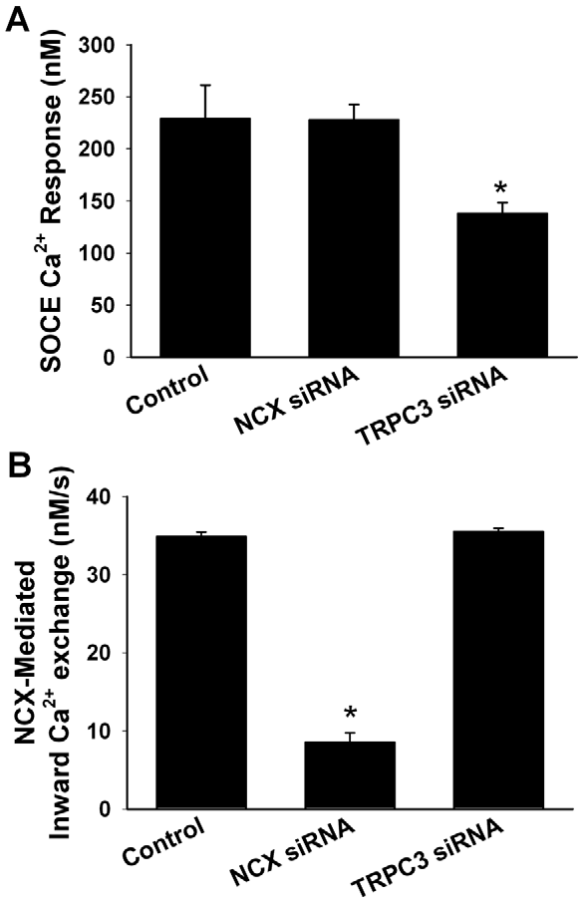
Relative contribution of SOCE vs. NCX to observed inward Ca^2+^ fluxes. (A) The extent of SOCE (evaluated using previously published protocols [37]) was substantially blunted by siRNA targeting one of the major mechanisms SOCE in human ASM (TRPC3) [37], but was unaffected by NCX1 siRNA. (B) In contrast, transfection of human ASM cells with NCX1 siRNA substantially inhibited inward Ca^2+^ exchange when evaluated using the protocol from Figure 3, supporting a role for NCX. However, siRNA targeting TRPC3 had no effect, demonstrating that the protocol largely elicits NCX within the measurement period. Values are means ± SE (n = 5 patient samples; minimum 70 cells per bar). * significant siRNA effect (p<0.05).

A potential concern with the influx protocol was the triggering of non-NCX inward Ca^2+^ fluxes upon re-introduction of extracellular Ca^2+^. We have previously demonstrated that SR store depletion in ASM leads to activation of SOCE [5] and in human ASM involves TRPC3 to a large extent [37] (with the understanding that other molecules such as STIM1 or Orai1 may also be involved [45]. While our protocol did not involve SR depletion, we wanted to confirm that any continuing SR Ca^2+^ leak in the presence of Xest-C or ryanodine was not a confounding factor. Therefore, in separate sets of cells, we tested the effect of TRPC3 siRNA on the presumed inward mode NCX Ca^2+^ changes, and conversely the effect of NCX siRNA on Ca^2+^ influx via the SOCE protocol [5]. We found that in cells transfected with NCX siRNA, the inward Ca^2+^ exchange in response to the SOCE protocol was not significantly different from non-transfected controls, whereas in cells transfected with TRPC3 siRNA, Ca^2+^ influx in the SOCE protocol was significantly blunted as expected (p<0.05), and consistent with previous studies [37]. In contrast to these observations, the rate of inward Ca^2+^ exchange following perfusion with zero-Na (normal Ca^2+^) Tyrodes in Na-loaded cells was significantly smaller in NCX siRNA transfected cells, but unaffected by TRPC3 siRNA (Figure 4), confirming that the inward mode NCX protocol used in this study predominantly reflects a Ca^2+^ influx via NCX and not via store-operated mechanisms.

**Figure 5:**
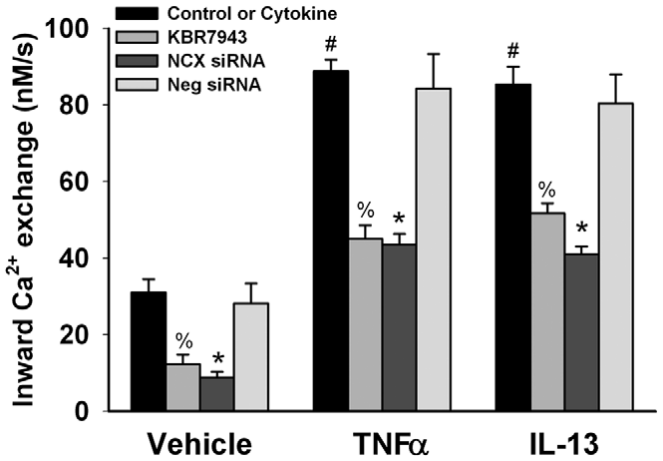
Effect of pro-inflammatory cytokines on inward Ca^2+^ exchange via NCX in human ASM. In fura-2 loaded ASM cells where this mode of NCX was evaluated using the protocol illustrated in Figure 3, the NCX inhibitor KBR7943 significantly inhibited inward Ca^2+^ exchange. Transfection with NCX1 siRNA also substantially inhibited inward exchange (while negative or scrambled siRNA had no significant effect). Exposure to TNFα or IL-13 (24 h) significantly enhanced inward exchange mode NCX compared to controls not exposed to cytokines. These cytokine-induced effects were blunted by KBR7943 or NCX1 siRNA (but not negative siRNA). Vehicle is culture media, HBSS, or Lipofectamine as appropriate. Values are means ± SE (n = 5 patient samples for controls, 3 patient samples for cytokine groups; minimum 60 cells per bar). % indicates significant KBR7943 effect, * significant NCX1 siRNA effect, and # significant cytokine effect (p<0.05).

We further verified NCX-mediated influx using KBR7943. In control ASM cells, following a thorough washout with normal Tyrodes solution for at least 20 min, pre-exposure to KBR7943 significantly blunted the rate (and magnitude) of Ca^2+^ inward exchange using the protocol described above (p<0.05; Figure 5). Similarly, in ASM cells transfected with NCX siRNA, the rate of inward Ca^2+^ exchange following perfusion with zero-Na Tyrodes in Na-loaded cells was significantly smaller (p<0.05; Figure 5). Transfection with negative (scrambled) siRNA did not substantially influence influx mode NCX, compared to Lipofectamine controls (Figure 5).

In ASM cells exposed for 24 h to TNFα or IL-13, baseline [Ca^2+^]_i_ levels were comparable to those of controls. In these cytokine-exposed cells, following Na-loading using the protocol above, perfusion with zero-Na Tyrodes resulted in significantly faster inward Ca^2+^ exchange compared to controls (p<0.05; Figure 5). The effects of TNFα and IL-13 on NCX-mediated inward Ca^2+^ exchange were comparable. Additionally, in ASM cells transfected with NCX siRNA or pre-exposed to KBR7943, the enhanced inward Ca^2+^ exchange due to TNFα and IL-13 were significantly blunted (p<0.05; Figure 5). Transfection with negative (scrambled) siRNA did not influence cytokine-induced enhancement of influx mode NCX (Figure 5).

**Figure 6:**
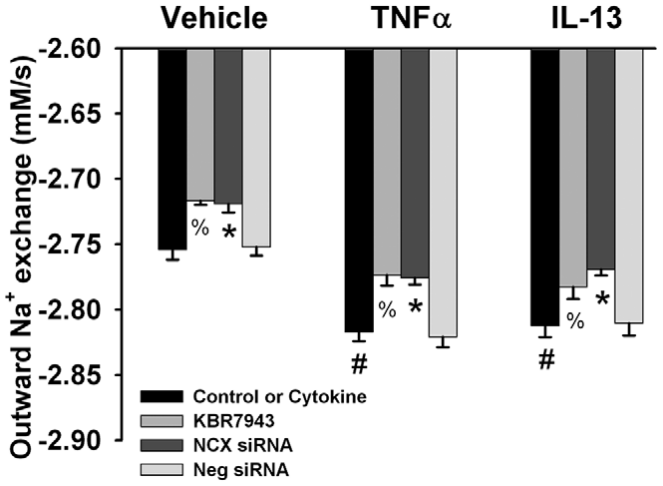
Effect of pro-inflammatory cytokines on Na^+^ outward exchange via NCX in human ASM. In SBFI loaded ASM cells where inward exchange-mode NCX was evaluated using the protocol illustrated in Figure 3, TNFα or IL-13 significantly enhanced Na^+^ outward exchange compared to controls not exposed to cytokines. KBR7943 and NCX1 siRNA (but not negative siRNA) both significantly inhibited outward Na^+^ exchange and blunted cytokine effects. Vehicle is culture media, HBSS, or Lipofectamine as appropriate. Values are means ± SE (n = 5 patient samples for controls, 3 patient samples for cytokine groups; minimum 50 cells per bar). % indicates significant KBR7943 effect, * significant NCX1 siRNA effect, and # significant cytokine effect (p<0.05).

In parallel studies using SBFI-loaded control ASM cells (to measure [Na^+^]_i_), initial [Na^+^]_i_ levels (based on SBFI calibrations) was ∼10 mM. A slow increase in [Na^+^]_i_ levels is observed with removal of Ca^2+^ using zero-Ca Tyrodes (with SR inhibition) (Figure 3; reflecting Na^+^ influx potentially through different mechanisms including outward Ca^2+^ exchange mode NCX). Under these conditions, estimated “peak” [Na^+^]_i_ levels did not exceed 25 mM. Subsequent rapid perfusion with zero-Na Tyrodes resulted in a rapid decrease in [Na^+^]_i_ levels (mirroring the rise in [Ca^2+^]_i_ levels; see Figure 3). The rate of fall in [Na^+^]_i_ in SBFI-loaded ASM cells transfected with NCX siRNA was significantly smaller compared to controls (p<0.05; Figure 6). In contrast, the rate of fall in [Na^+^]_i_ was significantly enhanced by exposure to TNFα or IL-13 for 24 h and significantly reduced by KBR7943 or NCX siRNA pretreatment (p<0.05; Figure 6; comparable between the two cytokines). As with the [Na^+^]_i_ measurements, negative (scrambled) siRNA did not significantly influence changes in [Na^+^]_i_ via NCX under control conditions, or the enhancing effect of cytokines (Figure 6).

### Outward Exchange Mode NCX

**Figure 7:**
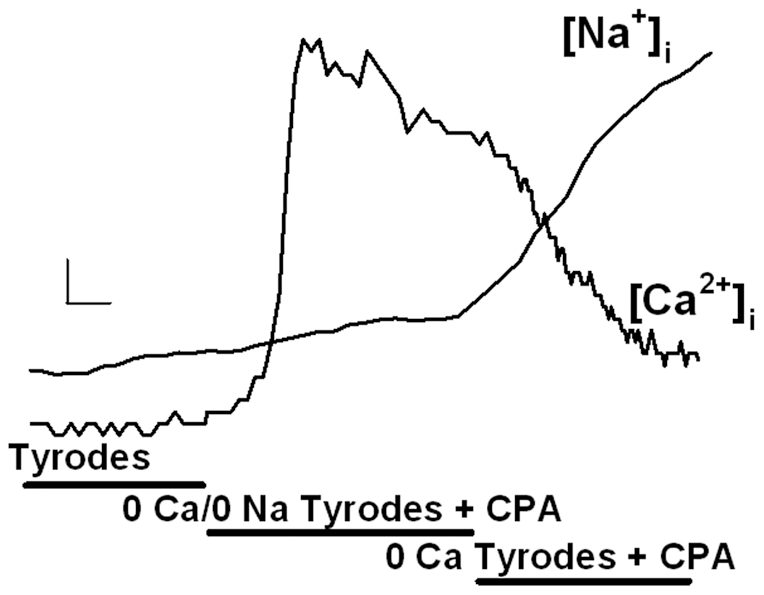
Evaluation of outward exchange mode NCX in human ASM cells using measurement of [Ca^2+^]_i_ (fura-2) vs. [Na^+^]_i_ (SBFI). Inhibition of SR Ca^2+^ reuptake using CPA in the absence of extracellular Ca^2+^ or Na^+^ (thus inhibiting NCX) resulted in increased [Ca^2+^]_i_ levels (with only small changes in [Na^+^]_i_ levels). Rapid re-introduction of extracellular Na^+^ resulted in activation of outward exchange-mode NCX, thus decreasing [Ca^2+^]_i_ while increasing [Na^+^]_i_. Horizontal scale bar is 10 s. Vertical scale bar is 50 nM for Ca^2+^ and 2 mM for Na^+^.

In control fura-2 loaded ASM cells perfused with normal Tyrodes solution, replacement of the perfusate with zero-Na, zero-Ca Tyrodes (along with 10 µM CPA) increased [Ca^2+^]_i_ levels (due to continued SR Ca^2+^ leak in the absence of reuptake), which eventually reached a plateau and then decreased slowly (likely reflecting continued efflux via plasma membrane Ca^2+^ ATPase). Rapidly reintroduction of Na^+^ (zero-Ca) Tyrodes resulted in a rapid decrease in [Ca^2+^]_i_ levels (Figure 7). The initial rate of fall of [Ca^2+^]_i_ ranged from 11.5 to 73.3 nM s^−1^ in control cells.

**Figure 8:**
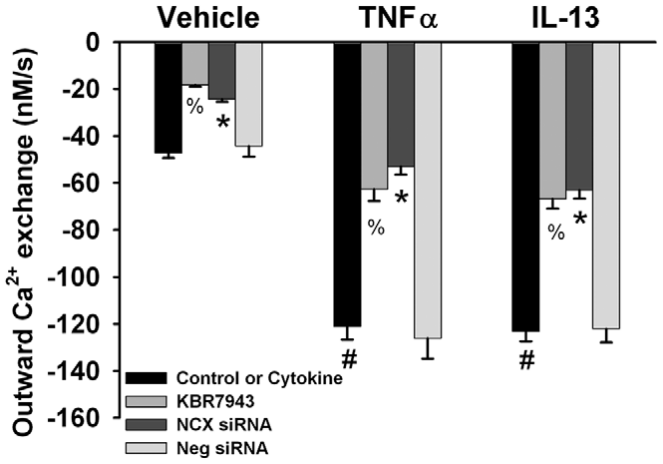
Effect of cytokines on outward Ca^2+^ exchange via NCX in human ASM. In fura-2 loaded ASM cells where outward exchange mode NCX was evaluated using the protocol illustrated in Figure 7, KBR7943 as well as NCX1 siRNA (but not negative siRNA) substantially inhibited outward Ca^2+^ exchange. Exposure to TNFα or IL-13 (24 h) significantly enhanced outward exchange-mode NCX (compared to controls not exposed to cytokines). KBR7943 and NCX1 siRNA also prevented cytokine-mediated increase in outward Ca^2+^ exchange. Values are means ± SE (n = 5 patient samples for controls, 3 patient samples for cytokine groups; minimum 60 cells per bar). % indicates significant KBR7943 effect, * significant NCX1 siRNA effect, and # significant cytokine effect (p<0.05).

In control ASM cells where outward exchange mode NCX was verified, following a thorough washout with normal Tyrodes solution for at least 20 min, pre-exposure to KBR7943 slightly (but significantly) blunted the rate of outward Ca^2+^ exchange using the protocol described above (p<0.05; Figure 8). Transfection with NCX siRNA resulted in substantial slowing of the rate of outward Ca^2+^ exchange following perfusion with zero-Ca Tyrodes in the protocol above (p<0.05; Figure 8). Negative siRNA had no effect on outward mode NCX, compared to vehicle control (Figure 8).

Exposure to TNFα or IL-13 for 24 h resulted in significantly faster outward Ca^2+^ exchange compared to controls (p<0.05; Figure 8). The effects of TNFα and IL-13 on NCX-mediated outward Ca^2+^ exchange were comparable. Inhibition of NCX using KBR7943 or expression using NCX siRNA significantly blunted cytokine effects on the observed Ca^2+^ responses (p<0.05; Figure 8). Negative siRNA did not affect cytokine-induced enhancement of outward mode NCX (Figure 8).

**Figure 9:**
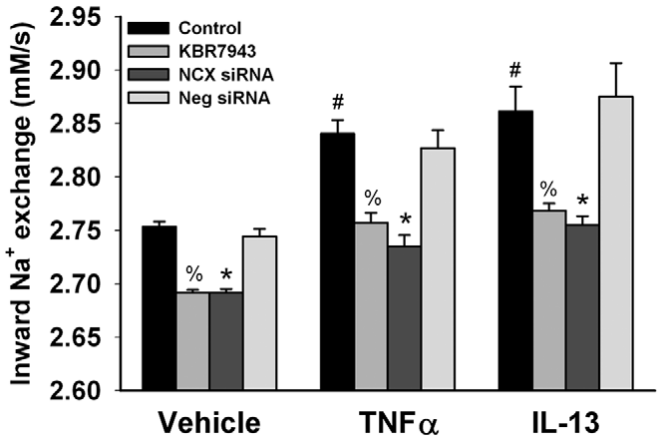
Effect of cytokines on inward Na^+^ exchange via NCX in human ASM. In SBFI loaded ASM cells where this mode of NCX was evaluated using the protocol illustrated in Figure 7, TNFα or IL-13 significantly enhanced inward Na^+^ exchange, while KBR7943 and NCX1 siRNA (but not negative siRNA) both significantly inhibited inward Na^+^ exchange. Values are means ± SE (n = 5 patient samples for controls, 3 patient samples for cytokine groups; minimum 50 cells per bar). % indicates significant KBR7943 effect, * significant NCX1 siRNA effect, and # significant cytokine effect (p<0.05).

In SBFI-loaded control ASM cells, exposure to zero-Na, zero-Ca Tyrodes (with CPA) did not significantly alter [Na^+^]_i_ levels compared to normal Tyrodes (Figure 7). Rapid reintroduction of zero-Ca Tyrodes resulted in a rapid increase in [Na^+^]_i_ levels. The rate of rise in [Na^+^]_i_ levels was substantially blunted in cells transfected with NCX siRNA (p<0.05; Figure 9). In contrast, exposure to TNFα or IL-13 substantially increased the rate of rise in [Na^+^]_i_ levels using the protocol above, the effect being inhibited by KBR7943 and NCX siRNA (p<0.05; Figure 9), but not by negative siRNA.

#### NCX and [Ca^2+^]_i_ Responses to Agonist

**Figure 10:**
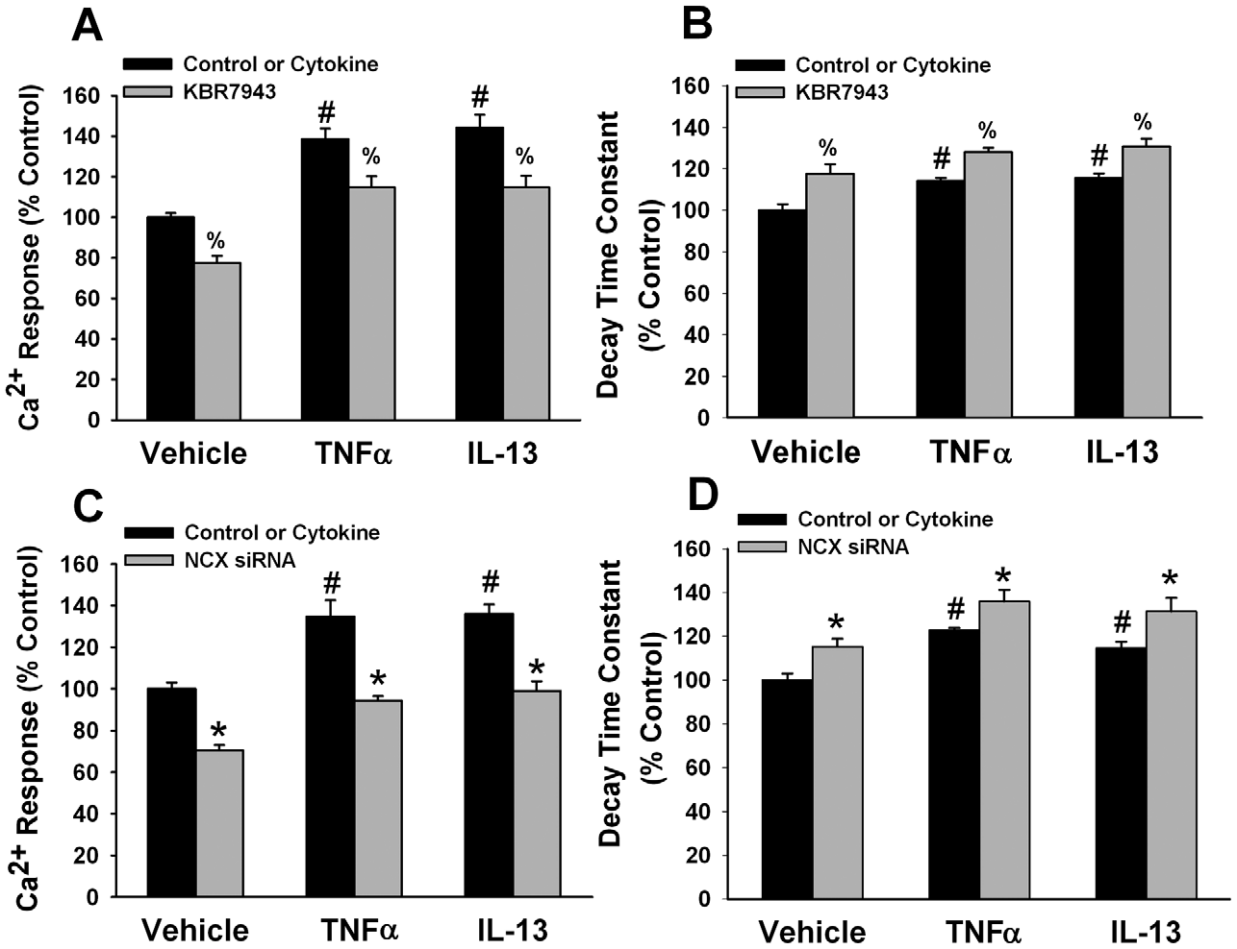
NCX in agonist-induced [Ca^2+^]_i_ responses. In ASM cells, 10 µM histamine stimulation resulted in the characteristic transient [Ca^2+^]_i_ response with high peak Ca^2+^ followed by decay to a lower plateau. (A) Peak [Ca^2+^]_i_ responses to histamine were significantly decreased by inhibition of NCX using KBR7943. In cells exposed to TNFα or IL-13, peak [Ca^2+^]_i_ responses were significantly increased, while KBR7943 blunted these effects. (B) The decay of [Ca^2+^]_i_ (fitted to single exponential with calculation of a time constant) was slowed by KBR7943 in cells exposed to vehicle, TNFα or IL-13; however these effects were smaller than KBR7943 effects on peak [Ca^2+^]_i_. Transfection with NCX1 siRNA also substantially blunted peak [Ca^2+^]_i_ (C) and slowed the decay of [Ca^2+^]_i_ responses (D) to histamine in vehicle- and cytokine-treated ASM cells. Values are means ± SE. (n = 5 patient samples for controls, 3 patient samples for cytokine groups; minimum 50 cells per bar). % indicates significant KBR7943 effect, * significant NCX1 siRNA effect, and # significant cytokine effect, (p<0.05).

Exposure of ASM cells to histamine resulted in the typical transient [Ca^2+^]_i_ response with an initial high peak (range 425–550 nM) which rapidly decayed to a lower plateau level (time constant of [Ca^2+^]_i_ decay 35–38 s based on a single exponential fit). Peak [Ca^2+^]_i_ was smaller and the rate of decay of the [Ca^2+^]_i_ response were both significantly slower in the presence of KBR7943 (p<0.05; Figure 10). Transfection with NCX1 siRNA also significantly affected these parameters (especially the peak [Ca^2+^]_i_ response), compared to Lipofectamine alone or negative siRNA (p<0.05; Figure 10). In cells exposed to TNFα or IL-13, peak [Ca^2+^]_i_ responses were substantially greater (800–900 nM for either cytokine), while the rate of fall of [Ca^2+^]_i_ was significantly slower (p<0.05; 44–49 s for either cytokine; Figure 10). Pre-exposure to KBR7943 or transfection with NCX siRNA significantly decreased peak [Ca^2+^]_i_ responses to histamine and slowed the rate of fall in [Ca^2+^]_i_ in cytokine-exposed cells. However, these effects were not proportionately greater than that observed in control cells. Furthermore, the effect of either KBR7943 or NCX siRNA on peak [Ca^2+^]_i_ was greater than that on the rate of fall of [Ca^2+^]_i_ (Figure 10).

## Discussion

In the present study, we demonstrate that human ASM cells express NCX protein, and display NCX-mediated inward Ca^2+^ exchange and outward Ca^2+^ exchange which contribute to [Ca^2+^]_i_ responses to agonist stimulation. These novel data point to a potentially rapid mechanism for regulating SR Ca^2+^ content in opposing ways: 1) providing Ca^2+^ via influx (i.e. inward Ca^2+^ exchange) mode, increasing [Ca^2+^]_i_ and facilitating SR refilling; or 2) removing Ca^2+^ via efflux mode (i.e. outward Ca^2+^ exchange), decreasing [Ca^2+^]_i_, and SR refilling. Furthermore, we found that the pro-inflammatory cytokines TNFα and IL-13, both known to enhance ASM [Ca^2+^]_i_ responses and airway contractility, increase NCX protein expression and enhance NCX-mediated Ca^2+^ fluxes. Thus, enhanced NCX expression and activity are potential mechanisms by which inflammation leads to increased airway contractility.

### NCX in smooth muscle

The role of NCX has been best established in cardiac muscle [15], [16], [17] and in neurons [46], [47], with efflux mode (outward Ca^2+^ exchange) being widely accepted [16], [18], [19]. However, under certain conditions (especially at the peak of the action potential) NCX-mediated Ca^2+^ influx can also occur in cardiac muscle, as demonstrated using inhibitors such as KBR7943 [16], [18], [19]. NCX regulation is certainly complex, with the relative contribution of different modes depending on a number of factors [15]. Furthermore, given substantial heterogeneity in NCX expression, function and regulation between tissues and species [15], it has been difficult to extrapolate physiological roles from cardiac muscle (for example) to smooth muscle. This is further made difficult by lack of specific NCX inhibitors (including KBR7943 itself [48]) and the wide variety of Ca^2+^ regulatory mechanisms in smooth muscle. Accordingly, these data are considerably less on NCX in smooth muscle.

Of the three major mammalian NCX isoforms, NCX1 has been shown to be expressed by a wide variety of tissues (including ASM) [15], [33], [49]. In smooth muscle tissues, NCX-mediated Ca^2+^ fluxes have been most well-established in vascular smooth muscle: a) NCX-mediated Ca^2+^ influx contributes to myogenic vasoconstriction in rat cremaster muscle arterioles [50]; b) in human umbilical artery, functional influx-mode NCX was inferred based on inhibitory effects of KBR7943 or decreased extracellular Na^+^ [51]; c) in porcine coronary artery [20], Ca uptake is affected by altered Na^+^ levels [52], and NCX is linked to SERCA, such that NCX-mediated Ca^2+^ influx facilitates SR Ca^2+^ refilling [53]. In this regard, the physical proximity of NCX to perimembranous SR [23], and a relationship between NCX and TRPC proteins [22], [25] further provide supporting evidence for NCX in [Ca^2+^]_i_ homeostasis. A recent electrophysiology based study suggests that reverse (or influx) mode NCX is active in ASM [33]. On the other hand, Schuster et al. found that in rat mesenteric artery, norepinephrine induced [Ca^2+^]_i_ oscillations were largely unaffected by KBR7943 (in contrast to inhibition of L-type Ca^2+^ channels) [54]. In other smooth muscles, NCX-mediated Ca^2+^ efflux appears to be important for [Ca^2+^]_i_ homeostasis in guinea pig stomach [55]. In urethral interstitial cells of Cajal, NCX-mediated Ca^2+^ influx is important for pacemaker activity [56]. Overall, these diverse studies generally support a role for both NCX-mediated Ca^2+^ influx and efflux in smooth muscle.

### [Ca^2+^]_i_ Regulation in ASM

It is now well-established that regulation of [Ca^2+^]_i_ in ASM involves SR Ca^2+^ release and reuptake as well as plasma membrane Ca^2+^ influx and efflux [1], [2], [4], [5], [12]. Ca^2+^ influx is known to occur through voltage-gated [10] and receptor-gated [11] channels, as well as in response to agonist-induced SR Ca^2+^ depletion (i.e. SOCE) [5], [12]. [Ca^2+^]_i_ homeostasis involves mechanisms to reduce Ca^2+^ levels. Here, the role of SR Ca^2+^ reuptake is well-established, and some studies have established that ASM expresses functional plasma membrane Ca^2+^ ATPases [31].

### NCX in ASM

Expression of NCX protein has been previously demonstrated in bovine and human ASM [28], [33]. Consistent with that finding, the present study also found that NCX1 is expressed in human ASM. Whether the other two mammalian NCX isoforms are expressed in ASM had not been examined previously. In this study, NCX2 and NCX3 protein levels were definitely undetectable, even following stimulation with cytokines. The detection of these NCX isoforms in rat brain extract, but not in ASM, underline their absence in ASM *per se*. These novel data suggest that even if the mRNA for these isoforms was present in human ASM (not examined in the current study, but suggested to be absent by Liu et al. [33]), only NCX1 is likely to play a functional role. Furthermore, several splice variants for NCX1 exist (resulting in molecular masses ranging from 110 to 160 kDa), which may differ in their relative sensitivities to Ca^2+^ or Na^+^ [15], [49]. In bovine ASM, protein bands at 160, 120 and 70 KDa were reported [27], [28]; however, in human ASM, the 160 KDa band appears to be absent in our experimental conditions. The functional implications of such variations in NCX expression remain to be determined.

Previous results regarding functional roles of NCX in ASM appear conflicting. NCX mediated Ca^2+^ influx may contribute to [Ca^2+^]_i_ in ASM of cow [28], pig [29], and guinea pig [30] but apparently not at all in dog [31]. The lack of NCX functionality in canine ASM noted more recently [31] is consistent with previous, older studies suggesting that NCX does not play a significant role in ASM [57], [58]. However, in guinea pig tracheal smooth rings as well as ASM cells stimulated with histamine, inhibition of Na^+^ influx or the use of KBR7943 decreased [Ca^2+^]_i_ and force, suggesting that nonspecific cation channels as well as NCX-mediated Ca^2+^ influx are indeed important in ASM force production [59]. Accordingly, the contribution of NCX in ASM may be species dependent. In this regard, the results of the present study in human ASM are novel. NCX-mediated inward Ca^2+^ exchange is evidenced by a) significant NCX1 protein expression; b) changes in inward Ca^2+^exchange (or equivalently outward Na^+^ exchange) in the presence of KBR7943 or with NCX1 siRNA; and c) reduction in the peak [Ca^2+^]_i_ response to histamine by both KBR7943 and NCX1 siRNA. Indeed, the significant NCX-mediated inward Ca^2+^ exchange that was noted in our study indicate a rapid mechanism for SR Ca^2+^ refilling, in addition to the recently established SOCE mechanism in ASM. As mentioned above, in vascular smooth muscle, it has been suggested that the proximity of NCX to perimembranous SR would allow for NCX-mediated influx to refill SR. Whether a similar situation occurs in ASM has not been directly demonstrated, but has been suggested by some investigators [27], [28]. In this regard, one potential mechanism for such interactions would be caveolar expression of NCX protein (demonstrated in other tissues [60]) wherein the plasma membrane invaginations would facilitate physical proximity to perimembranous SR, and thus SR Ca^2+^ refilling following NCX activation. Indeed, in pilot studies, we have found that NCX1 is expressed within caveolar fractions of human ASM cells (Pabelick, Thompson and Prakash, unpublished observations). Future studies will examine this important aspect of NCX function.

Whether efflux mode NCX contributes to [Ca^2+^]_i_ homeostasis in ASM is not clear. Even in vascular smooth muscle and other tissues where efflux mode NCX has been examined, interpretation may be limited by the fact that some were conducted in ASM tissues (i.e. not cells) where rapid changes in extracellular Ca^2+^ or Na^+^ required to verify NCX (typically <500 ms) cannot be satisfactorily achieved. Furthermore, pharmacological NCX inhibitors are not very specific [61]. Indeed, in our study, we found that KBR7943 equally affects both inward exchange and outward exchange modes of NCX, which is not surprising since other studies have also shown that at >1 µM, KBR7943 can inhibit either mode [61]. Approaches such as siRNA or viral transfection have not been applied to ASM. Finally, given that [Ca^2+^]_i_ sensitivity of inward exchange vs. outward exchange mode NCX differs [15], the contribution of these modes may differ between basal conditions, and extent of agonist stimulation. In the current study, we used human ASM cells and real-time imaging of [Ca^2+^]_i_ as well as intracellular Na^+^ ([Na^+^]_i_) to determine NCX fluxes. Using pharmacological inhibitors as well as siRNA, we determined that NCX-mediated outward exchange does make a significant contribution to [Ca^2+^]_i_ in human ASM, as evidenced by a) significant changes in outward Ca^2+^ exchange (or inward Na^+^ exchange) under conditions favoring NCX; b) significant effect of NCX1 siRNA; and c) the slowing of the decay of [Ca^2+^]_i_ responses to histamine. These findings represent a novel mechanism for decreasing [Ca^2+^]_i_ following agonist stimulation in addition to SR Ca^2+^ reuptake and plasma membrane Ca^2+^ ATPase. However, it must also be noted that the relative contribution of the outward vs. inward exchange modes of NCX to [Ca^2+^]_i_ regulation in ASM may not be the same: inhibition of NCX (either by KBR7943 or siRNA) appeared to have a greater effect on the peak [Ca^2+^]_i_ response to histamine (Figure 10) compared to the rate of fall of [Ca^2+^]_i_. This may represent the fact that following histamine stimulation, rapid NCX-mediated Ca^2+^ influx precedes the slower influx via mechanisms such as L-type channels or SOCE, while restoration of [Ca^2+^]_i_ levels may still be predominantly driven by SR reuptake.

### NCX and Inflammation

Although diseases such as asthma and chronic bronchitis are multifactorial in origin, the pathophysiology of such diseases has traditionally been correlated to an inflammatory process. Thus, the relevance of cytokine-induced changes in NCX1 expression lies in the potential contribution of this [Ca^2+^]_i_ regulatory mechanism to enhancing Ca^2+^ levels in inflamed ASM, as occurs in diseases such as asthma. There is currently no information on changes in NCX1 expression or activity in the diseased airway. Nonetheless, the results of the present study suggest that NCX1 expression and/or activity may be contributory in airway diseases.

While the list of cytokines potentially involved in asthma is long, both TNFα and IL-13 have been the focus of considerable investigation. TNFα has been shown to increase both agonist-induced [Ca^2+^]_i_ and force in ASM of several species including humans [6], [36], [37], [38]. Similarly, in mouse and rabbit trachea, IL-13 enhances the contractile response to cholinergic stimulation, while in the human, IL-13 increases changes in ASM stiffness induced by leukotrienes [39]. Based on these previous studies, we selected TNFα and IL-13 to examine the effect of inflammation on NCX in human ASM.

There is currently limited information on the effect of inflammation on NCX in any tissue. In cardiac muscle, early sepsis does not significantly alter NCX activity, but substantially blunts it during later stages [62]. In contrast, in rat microglia, interferon-gamma enhances NCX activity [63]. In human basophils, enhanced histamine release following IL-13 exposure has been attributed to increased NCX activity [64].

Recent reviews have suggested that Na^+^ may play a role in asthma [27], [28] and highlighted the existence of NCX in ASM. In the present study in human ASM, we found that both TNFα and IL-13 a) substantially increase NCX1 expression; and b) enhance NCX-mediated inward Ca^2+^ exchange as well as outward exchange. Regarding increased NCX1 expression, we further demonstrate that cytokine-induced effects are mediated via MEK1/2/ERK1/2 MAP kinases and NF-κB, both of which are important in enhanced [Ca^2+^]_i_ regulation. Whether increased NCX1 expression involves increased protein production, mRNA or protein stability has not been systematically examined previously. Our data using actinomycin D and cycloheximide suggest that cytokines do increase NCX1 transcription and new protein synthesis. Regardless, the effect of increased NCX1 protein expression is reflected by enhanced activity. Here, the asymmetric regulation of inward exchange vs. outward exchange modes may again be important, considering the finding that the two modes were affected to somewhat different extents by cytokine exposure. In addition to changing [Ca^2+^]_i_, cytokines may directly or indirectly depolarize the PM, change [Na^+^]_i_ gradients or affect ATP availability, all of which may affect the directionality of NCX. If the effects of inflammation lead to preferentially greater NCX-mediated inward exchange, [Ca^2+^]_i_ could be expected to increase, while [Ca^2+^]_i_ should decrease if outward exchange mode is affected more. However, such simple interpretations may be fallacious since cytokines can upregulate a number of other mechanisms that either increase or decrease [Ca^2+^]_i_ in ASM. Further studies are required to determine the overall effect of altered NCX on ASM contractility under conditions of inflammation. Regardless, our findings using KBR7943 and (especially) NCX siRNA suggest that upregulation of NCX expression and function may contribute to the altered ASM [Ca^2+^]_i_ regulation that occurs in airway inflammation.

### Methodological Issues

In this study, we used fluorescent dyes such as fura-2 and SBFI to measure [Ca^2+^]_i_ and [Na^+^]_i_ changes in human ASM cells. Empirical calibrations of the ionic concentrations were performed to allow assessment of relative changes with inflammation or with drug exposures. However, it must be noted that the usual 3∶1 Na^+^: Ca^2+^ molar stoichiometry of NCX cannot be easily derived using fluorescent techniques such as these since only the overall [Na^+^]_i_ (or [Ca^2+^]_i_) is being measured, rather than the dynamic peri-membranous ionic fluxes. Accordingly, calibrated signals provide values of nM Ca^2+^ (fura-2) but mM Na^+^ (SBFI). Therefore, we have used the terms “inward exchange” to represent NCX-mediated influx and “outward exchange” to represent NCX-mediated efflux, to highlight the fact that actual fluxes were not measured.

### Conclusions

In conclusion, in the present study, we demonstrate that human ASM cells express functional NCX1 protein with both inward exchange and outward exchange modes NCX contributing to [Ca^2+^]_i_ responses to agonist stimulation. These novel data point to a rapid mechanism for regulating [Ca^2+^]_i_ in ASM. The pro-inflammatory cytokines TNFα and IL-13, both known to enhance ASM [Ca^2+^]_i_ responses and airway contractility, increase NCX expression and enhance NCX-mediated Ca^2+^ fluxes. Thus, enhanced NCX expression and activity are potential mechanisms by which inflammation leads to increased airway contractility.
